# Supplementary material for: Barriers and enablers for the deployment of large language model-based conversational robots for older adults: A protocol for a systematic review of qualitative studies
Source: PLoS One. 2025 Apr 1;20(4):e0321093. doi: 10.1371/journal.pone.0321093 (PMC11960934; doi:10.1371/journal.pone.0321093)
Supplement: Attachment — Submitted filename: Response to Reviewers.docx [file pone.0321093.s002.docx]

**Response to Reviewer Comments and Manuscript Changes**

We thank both the Editor and reviewer for their thoughtful and constructive feedback on our manuscript. We have carefully addressed each point and made appropriate revisions to strengthen the protocol. Below we provide detailed responses to each comment.

**Response to Editor’s Comments**

| **S.No.** | **Reviewer Comment** | **Response** | **Changes Made in Manuscript** |
| --- | --- | --- | --- |
| 1. | We understand that the proposed inclusion criteria are restricted to studies published in the English language due to resource constraints, and that you have mentioned this as a potential limitation. Please could you update the discussion/limitation section of your manuscript with a more in-depth discussion of the potential consequences of the imposed language restriction, for example potential bias in reporting. | We acknowledge this limitation and have expanded our discussion to address potential biases and consequences of the language restriction. The updated text provides specific examples of how this might affect representation of non-English speaking regions and impact our findings. | Added detailed paragraph after line 349: "Furthermore, the restriction to English language publications may introduce language and geographic bias in our findings. This could particularly impact the representation of experiences and perspectives from non-English speaking regions, especially parts of Asia and Europe where there has been significant development and deployment of conversational AI technologies for older adults. Cultural nuances and context-specific implementation factors from these regions may be missed. Additionally, this language restriction could lead to publication bias since studies with significant findings from non-English speaking countries are more likely to be published in English, while studies with negative or null findings may only be published in local languages." |
| 2. | Please review your reference list to ensure that it is complete and correct. If you have cited papers that have been retracted, please include the rationale for doing so in the manuscript text, or remove these references and replace them with relevant current references. | We have conducted a thorough review of all references and confirmed their current status. No retracted papers were included in our reference list. We have added a statement confirming this verification process. | No changes required. |

**Response to Reviewer 1 Comments**

| **S.No.** | **Reviewer Comment** | **Response** | **Changes Made in Manuscript** |
| --- | --- | --- | --- |
| 1. | The protocol is well-written, comprehensive, and methodologically rigorous. It adheres to established guidelines such as PRISMA-P and ENTREQ, which enhances its transparency and reproducibility. | We thank the reviewer for their positive feedback. No specific changes were requested. | No changes required. |
| 2. | Although the databases are comprehensive, adding more databases that address engineering field may be helpful, especially there may be multiple pilot studies that have published in these journals that may include qualitative data about experience. | We agree with this suggestion and have expanded our database coverage to include key engineering and computer science databases. | Added new content after line 168: "Additional engineering and computer science databases including IEEE Xplore, ACM Digital Library, and Engineering Village will be searched to capture relevant technical studies and pilot implementations that may contain qualitative data about user experiences and implementation challenges. These databases were selected to ensure comprehensive coverage of human-computer interaction and robotics research involving conversational agents for older adults." |
| 3. | Consider discussing how you will handle grey literature, as relevant insights from unpublished studies or conference proceedings may add value to the review. | We have added a detailed description of our approach to including and handling grey literature, including specific sources and quality considerations. | Added new section after line 177: "Grey literature will be included through targeted searches of relevant conference proceedings, technical reports, theses/dissertations, and organizational websites. Sources will include major gerontechnology and human-robot interaction conferences, research institutions focused on aging and technology, and organizations involved in AI ethics and governance. Including grey literature will help capture emerging findings and reduce publication bias. However, grey literature will be clearly identified and considered separately in the quality assessment and confidence ratings." |
| 4. | Backward citation analysis using Google Scholar may be also helpful, beside using the manual search for the references lists. | We have incorporated this suggestion and added details about using Google Scholar for backward citation analysis. | Added new content after line 216: "In addition to manual reference list searching, backward citation analysis will be conducted using Google Scholar to identify additional relevant studies that have cited the included papers. This systematic citation tracking will help ensure comprehensive coverage of the literature and identify more recent related work that builds upon the primary studies." |

We believe these revisions have substantially strengthened the protocol while maintaining its core methodological rigor. The added details will enhance reproducibility and implementation. We welcome any additional feedback from the reviewers.
